# Application of the BL-BOPPPS Model in Electric Operating Bed Training for Operating Room Nurses

**DOI:** 10.5334/pme.1895

**Published:** 2026-05-05

**Authors:** Ji-ping Yang, Hua Xie, Yifeng Zhou, Hao Yuan

**Affiliations:** 1 Department of Operating, Hunan Provincial People’s Hospital (The First Affiliated Hospital of Hunan Normal University), Changsha 410005, China; 2 Department of Clinical laboratory, Hunan Provincial People’s Hospital, The First Affiliated Hospital of Hunan Normal University, Changsha, China

## Abstract

**Background:**

Operating room nurses (ORNs) require proficient electric operating bed (EOB) skills to ensure patient safety and surgical efficiency. The Bridge-in, Learning Outcomes, Pre-assessment, Participatory learning, Post-assessment, and Summary (BOPPPS) model integrated with blended learning (BL) represents an innovative educational approach, but its effectiveness in EOB training remains unexplored.

**Methods:**

A randomized controlled trial was conducted with 400 ORNs randomly assigned to either an experimental group receiving BL-BOPPPS-based EOB training (n = 200) or a control group receiving traditional training (n = 200). Both groups participated in two 90-minute sessions. Outcome measures included EOB operation skills (primary outcome), self-efficacy, and satisfaction (secondary outcomes). Assessments were conducted at pre-intervention, immediately post-intervention, and at 3-month follow-up.

**Results:**

The experimental group demonstrated significantly higher improvements in EOB operation skills (mean difference: 18.74 points, 95% CI: 15.92–21.56, p < 0.001) and self-efficacy (mean difference: 9.63 points, 95% CI: 8.21–11.05, p < 0.001) compared to the control group. The experimental group also reported significantly higher satisfaction scores (mean difference: 5.87 points, 95% CI: 5.12–6.62, p < 0.001). Multiple regression analysis identified participation in the BL-BOPPPS model, years of OR experience, and baseline self-efficacy as significant predictors of EOB operation skills improvement. These improvements were maintained at the 3-month follow-up in the experimental group.

**Conclusion:**

The BL-BOPPPS model represents an effective approach for EOB training among ORNs, producing superior improvements in operation skills, self-efficacy, and satisfaction compared to traditional training methods. These findings suggest that integrating this pedagogical model into clinical nursing education could enhance the effectiveness of technical skills training.

Clinical Trial Registration

ID: ISRCTN34671997

https://www.isrctn.com/ISRCTN34671997

## Introduction

Operating room nurses (ORNs) play a crucial role in ensuring patient safety and surgical efficiency, requiring specialized technical skills and knowledge to navigate the complex environment of modern operating rooms [1]. Among these essential skills, proficient operation of electric operating beds (EOBs) is particularly critical, as it directly impacts patient positioning, surgical access, and prevention of pressure injuries during procedures. With the increasing technological complexity of EOBs and their central role in surgical procedures, effective training methodologies for ORNs have become increasingly important [2].

Traditional approaches to EOB training typically rely on didactic lectures, demonstrations, and limited hands-on practice, often failing to prepare nurses adequately for the dynamic challenges of the operating room environment [3]. These conventional methods, characterized by passive learning and limited interaction, have been associated with suboptimal skill acquisition, reduced knowledge retention, and diminished transfer to clinical practice [4]. The shortcomings of traditional training approaches are particularly concerning in the context of operating room safety, where technical errors in EOB operation can lead to patient injuries, surgical complications, and workflow disruptions.

The need for more effective educational strategies has prompted an exploration of innovative pedagogical models in nursing education. Several participatory, constructivist instructional approaches have been proposed in the literature. For example, van Merriënboer and Kirschner [5] developed the Four-Component Instructional Design (4C/ID) model, which organizes complex learning around whole-task practice, supportive information, procedural information, and part-task practice. While 4C/ID has demonstrated effectiveness in various professional training contexts, its implementation requires extensive task analysis and considerable curriculum development time, which can be challenging in rapidly evolving clinical environments. Other models, such as problem-based learning [6] and team-based learning, have also shown promise but may lack the structured sequential framework desirable for time-limited technical skills training.

The Bridge-in, Learning Outcomes, Pre-assessment, Participatory learning, Post-assessment, and Summary (BOPPPS) model represents an alternative approach that offers a structured yet practical framework emphasizing learner engagement, active participation, and formative assessment [7]. Originally developed in higher education contexts, the BOPPPS model provides a comprehensive instructional design that addresses various aspects of the learning process: activating prior knowledge, clarifying learning objectives, assessing baseline understanding, facilitating active learning, evaluating learning outcomes, and synthesizing key concepts [8]. Compared to other participatory approaches, the BOPPPS model is particularly well-suited for structured clinical skills training because of its modular design, which allows each instructional session to be self-contained and systematically evaluated.

The design of the BL-BOPPPS intervention in the present study was guided by several complementary learning theories. First, constructivist learning theory posits that learners actively construct knowledge through meaningful experiences and reflection rather than passively receiving information [9,10]. In the BL-BOPPPS model, this principle is operationalized through the Participatory Learning phase, which engages learners in hands-on practice, problem-solving, and peer interaction to build understanding through direct experience. Second, Vygotsky’s sociocultural theory and the concept of the zone of proximal development (ZPD) emphasize that higher-order cognitive functions develop through social interaction and that learning is most effective when it occurs within the gap between what learners can accomplish independently and what they can achieve with guidance [11]. The BL-BOPPPS model incorporates this principle through structured instructor demonstrations, peer collaboration, and graduated feedback during the participatory learning phase. Third, adult learning principles, as articulated by Knowles and further developed by subsequent scholars, highlight the importance of self-direction, relevance, and problem-centeredness in adult education [12,13]. The blended learning component of the intervention addresses these needs by providing flexible, self-paced online resources that allow learners to prepare independently before face-to-face sessions. Finally, situated cognition theory posits that learning is inherently tied to authentic contexts and activities, suggesting that training should simulate real-world conditions to facilitate knowledge transfer [14,15]. The use of simulated operating room environments and realistic clinical scenarios in the BL-BOPPPS training was explicitly designed to align instruction with the situated demands of clinical practice. Together, these theoretical frameworks provided the conceptual foundation for the selection and integration of each component of the BL-BOPPPS intervention.

In recent years, the BOPPPS model has been integrated with blended learning (BL) strategies, creating a hybrid approach that combines the benefits of face-to-face instruction with online learning components [16]. This BL-BOPPPS model leverages digital technologies to enhance the flexibility, accessibility, and interactivity of training, while maintaining the structured pedagogical framework of the BOPPPS approach [17]. The integration of digital resources, such as videos, interactive simulations, and online assessments, expands the reach of traditional training and provides opportunities for self-paced learning and immediate feedback.

Preliminary research suggests that the BL-BOPPPS model may offer significant advantages over traditional educational approaches in various healthcare education contexts. Ma et al. [18] found that implementing the BL-BOPPPS model in health services management education resulted in improved student outcomes and greater learner satisfaction compared to conventional teaching methods. Similarly, Dai et al. [19] reported enhanced knowledge acquisition and critical thinking skills among veterinary medicine students taught using the BL-BOPPPS approach. However, the effectiveness of this model in nursing education, particularly in technical skills training for specialized nursing roles, remains largely unexplored.

The operating room environment presents unique educational challenges that may be particularly amenable to the BL-BOPPPS approach. The high-stakes, technology-intensive nature of operating room nursing requires educational methods that can effectively bridge theoretical knowledge and practical application [20]. Moreover, the increasing technological sophistication of EOBs demands training approaches that can adapt to evolving equipment designs and functionalities. The structured yet flexible nature of the BL-BOPPPS model may provide an ideal framework for addressing these challenges, offering a systematic approach to skill development while accommodating the dynamic realities of clinical practice.

Previous studies have demonstrated the importance of effective EOB training for ORNs. Kim and Park [4] found that implementing a simulation-based educational program significantly improved nurses’ knowledge, self-efficacy, and performance in operating electric beds. Similarly, McRae et al. [21] reported high levels of effectiveness and learner satisfaction with simulation-based training for cardiac surgical skills among nurses. These findings suggest that interactive, simulation-based approaches may be superior to traditional methods for technical skills training in nursing education.

However, despite the promising potential of the BL-BOPPPS model for EOB training, empirical evidence regarding its effectiveness in this specific context is lacking. While the model has demonstrated success in other educational settings, its application to specialized nursing skills, particularly in the operating room environment, has not been systematically evaluated. This research gap limits the evidence base for educational practices in nursing, potentially hindering the adoption of innovative pedagogical approaches that could enhance the quality and efficiency of training.

Additionally, the relationships between the BL-BOPPPS model and learning outcomes in EOB training remain underexplored. Understanding which learner characteristics and training components are associated with skill improvement is essential for optimizing training programs and tailoring educational interventions to the specific needs of ORNs. By examining the relationship between participation in the BL-BOPPPS model and various learning outcomes, including technical skills, self-efficacy, and satisfaction, this study aims to provide insights into the factors that contribute to effective learning in this context.

The present study addresses these gaps by investigating the application of the BL-BOPPPS model in EOB training for ORNs. By comparing this innovative approach to traditional training methods, the study seeks to provide empirical evidence regarding the effectiveness of the BL-BOPPPS model for enhancing EOB operation skills, self-efficacy, and learner satisfaction among ORNs. Furthermore, by examining the factors that influence learning outcomes in this context, the study aims to contribute to a deeper understanding of effective educational practices in specialized nursing education.

The findings of this study have potential implications for nursing education, clinical practice, and patient safety. If the BL-BOPPPS model proves effective for EOB training, it could inform the development of educational programs for other technical skills in nursing, potentially enhancing the quality and efficiency of nursing education more broadly. Moreover, by improving EOB operation skills among ORNs, the study may indirectly contribute to enhanced patient safety, surgical efficiency, and overall quality of care in the operating room environment.

Therefore, the purpose of this study was to investigate the effectiveness of the BL-BOPPPS model in EOB training for ORNs compared to traditional training methods. The specific objectives were to: (1) compare the effects of BL-BOPPPS-based training and traditional training on EOB operation skills among ORNs; (2) examine the impact of the BL-BOPPPS model on ORNs’ self-efficacy and satisfaction with training; and (3) identify factors associated with the effectiveness of EOB training using the BL-BOPPPS approach.

## Methods

### Study Design

This study adopted a randomized controlled trial design and was conducted from March 2022 to May 2022 in Chang Sha. The participants were randomly assigned to either the experimental group or the control group. The experimental group received the EOB training based on the BL-BOPPPS model, while the control group received the traditional EOB training. The study was conducted in a simulated operating room in a nursing school. The study protocol was approved by the Ethics Committee of our hospital (ID: 2021 SRLR No.93) and the participants gave informed consent before the study. All procedures were performed in accordance with the ethical standards of the institutional and national research committee and with the 1964 Helsinki declaration and its later amendments.

A power analysis was conducted to determine the required sample size. Based on previous similar studies, we anticipated a medium effect size (Cohen’s d = 0.5) for the primary outcome measure. With an alpha level of 0.05 and a power of 0.8, the required sample size was calculated to be 64 participants per group. Considering a potential attrition rate of 20%, we aimed to recruit 80 participants per group, for a total sample of 160 participants. However, to enhance the robustness of our findings and allow for potential subgroup analyses, we decided to recruit 200 participants per group, for a total sample of 400 participants.

### Participants

The participants were 400 ORNs who worked in tertiary hospital in Changsha, China. The inclusion criteria were: (1) having at least one year of working experience in the operating room; (2) having no prior EOB training or certification; and (3) being willing to participate in the study. The exclusion criteria were: (1) having any physical or mental conditions that could affect the learning process, specifically musculoskeletal disorders limiting the ability to perform hands-on bed operations, visual or hearing impairments not corrected by assistive devices, and self-reported psychiatric conditions (e.g., major depression or anxiety disorder) currently under active treatment; these conditions were identified through a brief standardized health screening questionnaire administered during the eligibility assessment; and (2) being absent from the training or the assessment sessions.

A total of 468 ORNs were initially assessed for eligibility, of whom 400 met the inclusion criteria and were enrolled in the study. The participants were randomly allocated to the experimental group (n = 200) or the control group (n = 200) using a computer-generated random number table. The randomization sequence was generated by a statistician who was not involved in the recruitment or assessment of the participants. The allocation was concealed from the researchers who recruited the participants until the interventions were assigned.

The demographic characteristics of the participants, including age, gender, education level, years of working experience in the operating room, and previous training experience, were collected using a self-administered questionnaire before the training. The baseline characteristics were compared between the two groups to ensure the effectiveness of the randomization.

### Intervention

#### Experimental Group: BL-BOPPPS Model Training

The experimental group received EOB training based on the BL-BOPPPS model. The training consisted of two sessions, each lasting for 90 minutes, with a one-week interval between sessions. The first session covered the basic knowledge and operation of the EOB, including the structure, function, and safety precautions. The second session covered the advanced operation of the EOB, including the adjustment of the patient’s position and angle according to surgical needs.

The blended learning component of the intervention involved a combination of online and face-to-face elements. Prior to the face-to-face sessions, participants were given access to an online learning platform that contained instructional videos, interactive tutorials, and knowledge quizzes related to EOB operation. These resources were designed to provide foundational knowledge and prepare participants for the hands-on components of the training. Participants were required to complete the online modules at least three days before each face-to-face session. Access to the online platform remained available to participants throughout the study period, including after completion of the face-to-face sessions, allowing them to review materials at any time. Completion of the online modules was monitored through the platform’s learning management system. Participants who did not complete the required modules within the specified timeframe were contacted by the research team and given additional time to complete the modules before the face-to-face session; however, no participants were excluded from the study for late completion, and this factor did not contribute to any observed attrition.

During the face-to-face sessions, the BOPPPS model was applied as follows:

**Bridge-in (10 minutes):** The instructors introduced the topic and objectives of the training, activating the learners’ prior knowledge and motivation through targeted questions, video demonstrations of EOB-related incidents, and clinical case scenarios that highlighted the importance of proper EOB operation.

**Learning Outcomes (5 minutes):** The instructors clearly stated the specific and measurable learning outcomes for the session, explaining how these outcomes would be assessed and evaluated. Learning outcomes were presented both in a projected slide listing the session objectives and through verbal explanation by the instructor, with explicit connections to clinical practice requirements.

**Pre-assessment (15 minutes):** Participants’ baseline knowledge and skills were assessed using a combination of multiple-choice questions, short-answer questions, and simple demonstration tasks. This pre-assessment served to identify knowledge gaps and tailor the subsequent instruction to the learners’ needs. Immediate feedback was provided on the pre-assessment performance.

**Participatory Learning (40 minutes):** This core component involved active engagement with the content through varied learning activities. Participants practiced EOB operations in pairs or small groups, engaged in problem-solving exercises based on clinical scenarios, participated in simulation exercises, and received immediate feedback from instructors and peers. The instructors used a combination of demonstration, guided practice, and independent practice to facilitate skill development.

**Post-assessment (15 minutes):** Participants’ knowledge and skills were reassessed using methods similar to the pre-assessment but with different questions and tasks of equivalent difficulty. This allowed for measurement of learning gains and identification of areas requiring further instruction or practice. The instructors provided detailed feedback on performance and addressed any persistent misconceptions.

**Summary (5 minutes):** The instructors summarized the key points and learning outcomes of the session, reinforcing the critical aspects of EOB operation. Participants were encouraged to reflect on their learning experience, articulate insights gained, and identify areas for further improvement. Connections to clinical applications were emphasized to promote transfer of learning.

The training was delivered by two experienced ORNs who were trained in both the BL-BOPPPS model and EOB operation. These instructors had a minimum of five years of operating room experience and had completed a 40-hour training program on the BL-BOPPPS teaching methodology. The instructor-to-participant ratio was maintained at 1:10 to ensure adequate supervision and feedback during the hands-on components of the training. Given that there were 200 participants in the experimental group and the instructor-to-participant ratio was 1:10, each 90-minute session was conducted 10 times with groups of approximately 20 participants (10 per instructor). The same 20 participants were assigned to a consistent group for both sessions to maintain group cohesion and facilitate progressive skill building. Group compositions were predetermined by the randomization schedule and remained stable across the two training sessions.

#### Control Group: Traditional Training

The control group received traditional EOB training, which also consisted of two sessions, each lasting for 90 minutes, with a one-week interval between sessions. The content coverage was identical to that of the experimental group, addressing both basic and advanced aspects of EOB operation.

The traditional training followed a teacher-centered approach, primarily consisting of didactic lectures (approximately 60 minutes per session) and instructor demonstrations (approximately 30 minutes per session). Participants primarily engaged as passive recipients of information, with limited opportunities for hands-on practice or active participation. Questions were addressed at the end of each session, but structured feedback on performance was minimal.

The traditional training was delivered by two experienced ORNs who were not trained in the BL-BOPPPS model but had equivalent clinical experience and expertise in EOB operation as the instructors in the experimental group. These instructors had not been exposed to the BL-BOPPPS methodology to prevent contamination between the groups. Standardized lesson plans and training materials were developed collaboratively by the research team and provided to the control group instructors to ensure that the content and depth of coverage matched those of the experimental group, with the only systematic difference being the pedagogical approach.

### Outcome Measures

#### Primary Outcome: EOB Operation Skills

The primary outcome was the EOB operation skills of the participants, measured by a standardized EOB operation skill test. This test was developed and validated by a panel of five experts, including two senior ORNs, two nurse educators, and one anesthesiologist, all with at least 10 years of operating room experience.

The test consisted of 20 items, each scored from 0 to 5, with a total possible score of 100. A higher score indicated a higher level of EOB operation skills. The items covered both basic operations (e.g., adjusting the height and lateral tilt of the bed) and advanced operations (e.g., configuring the bed for specific surgical positions such as lithotomy, Trendelenburg, and reverse Trendelenburg). The test also assessed safety considerations, efficiency of movement, and adaptability to different clinical scenarios.

The test was administered by two trained raters who were blinded to the group allocation of the participants. The raters were experienced ORNs who had not been involved in the delivery of either training program. Before the study, the raters underwent a standardization training to ensure consistent evaluation. The inter-rater reliability of the test was calculated using the intraclass correlation coefficient (ICC), which was 0.92, indicating excellent reliability.

The EOB operation skill test was conducted at three time points: before the training (baseline), immediately after the training (post-intervention), and three months after the training (follow-up). The follow-up assessment was included to evaluate the retention of skills over time.

#### Secondary Outcomes

**Self-efficacy:** The participants’ self-efficacy regarding EOB operation was measured using a 10-item EOB Operation Self-Efficacy Scale, adapted from the General Self-Efficacy Scale [22]. The scale asked participants to rate their confidence in performing various EOB operations on a 5-point Likert scale, ranging from 1 (not at all confident) to 5 (very confident). The total score ranged from 10 to 50, with higher scores indicating greater self-efficacy. Example items included: “I am confident in my ability to adjust the EOB for different surgical positions” and “I can troubleshoot common EOB problems effectively during surgery.” The scale demonstrated good internal consistency with a Cronbach’s alpha of 0.88. Self-efficacy was measured at the same three time points as the primary outcome: baseline, post-intervention, and three-month follow-up.

**Satisfaction:** Participants’ satisfaction with the training was assessed using a 5-item EOB Training Satisfaction Scale, developed based on Kirkpatrick’s model of training evaluation [23]. The scale asked participants to rate their satisfaction with various aspects of the training on a 5-point Likert scale, ranging from 1 (very dissatisfied) to 5 (very satisfied). The aspects evaluated included the content, teaching methods, instructors, learning environment, and overall experience. The total score ranged from 5 to 25, with higher scores indicating greater satisfaction. The scale demonstrated good internal consistency with a Cronbach’s alpha of 0.89. Satisfaction was measured only at post-intervention.

### Data Collection Procedures

Data collection was conducted by research assistants who were not involved in the delivery of the interventions and were blinded to the group allocation of the participants. The baseline assessment, including demographic information, EOB operation skills, and self-efficacy, was conducted one week before the first training session. The post-intervention assessment, including EOB operation skills, self-efficacy, and satisfaction, was conducted one week after the second training session. The follow-up assessment, including EOB operation skills and self-efficacy, was conducted three months after the completion of the training.

All assessments were conducted in the same simulated operating room where the training took place, using the same model of EOB to ensure consistency. The EOB operation skill test was video-recorded for later evaluation by the blinded raters. The participants completed the self-efficacy and satisfaction scales independently, without discussion with other participants or the researchers.

### Data Analysis

Statistical analyses were performed using SPSS version 25.0 (IBM Corp., Armonk, NY, USA). The normality of the data was assessed using the Shapiro-Wilk test. Descriptive statistics, including means, standard deviations, frequencies, and percentages, were calculated for the demographic characteristics and outcome variables. Baseline comparisons between the two groups were conducted using independent t-tests for continuous variables and chi-square tests for categorical variables.

For the primary outcome analysis, a mixed-design analysis of variance (ANOVA) was used to compare the changes in EOB operation skills between the two groups across the three time points (baseline, post-intervention, and follow-up). Post-hoc analyses with Bonferroni correction were conducted for significant interaction effects. Cohen’s d was calculated to estimate the effect size of the between-group differences.

Similar analyses were performed for the self-efficacy outcome. For the satisfaction outcome, which was measured only at post-intervention, an independent t-test was used to compare the difference between the two groups.

Multiple linear regression analysis was conducted to identify factors that predicted EOB operation skills improvement (defined as the difference between post-intervention and baseline scores). The predictors included in the model were group allocation (experimental vs. control), age, gender, education level, years of working experience in the operating room, baseline self-efficacy, and baseline EOB operation skills.

The relationship between self-efficacy and EOB operation skills was examined using Pearson’s correlation coefficient. A receiver operating characteristic (ROC) curve analysis was performed to determine the optimal cut-off value of self-efficacy for predicting successful acquisition of EOB operation skills (defined as achieving at least 80% of the maximum score on the EOB operation skill test).

Missing data were handled using the multiple imputation method. A sensitivity analysis was performed to assess the robustness of the findings by comparing the results of the complete-case analysis with those of the multiple imputation analysis. All statistical tests were two-tailed, with a significance level of 0.05.

## Results

### Participant Characteristics

A total of 468 ORNs were initially assessed for eligibility, of whom 400 met the inclusion criteria and were enrolled in the study. Among the 68 excluded ORNs, 35 had prior EOB training, 25 had less than one year of working experience in the operating room, and 8 declined to participate. All 400 enrolled participants completed the baseline and post-intervention assessments, resulting in a completion rate of 100%. For the three-month follow-up assessment, 12 participants (6 from each group) were lost to follow-up, resulting in a retention rate of 97%.

Table 1 presents the baseline demographic and clinical characteristics of the participants. There were no significant differences between the experimental and control groups in terms of age, gender, education level, years of working experience, or baseline measures of EOB operation skills and self-efficacy, indicating successful randomization.

**Table 1:** Baseline Characteristics of Participants.


CHARACTERISTIC	EXPERIMENTAL GROUP (n = 200)	CONTROL GROUP (n = 200)	P-VALUE

**Age (years), mean ± SD**	31.45 ± 6.82	30.98 ± 7.13	0.631

**Gender, n (%)**			0.749

Female	166 (83.0)	170 (85.0)	

Male	34 (17.0)	30 (15.0)	

**Education level, n (%)**			0.873

Diploma	24 (12.0)	20 (10.0)	

Bachelor’s degree	156 (78.0)	162 (81.0)	

Master’s degree or higher	20 (10.0)	18 (9.0)	

**Years of working experience in OR, mean ± SD**	5.73 ± 3.95	5.48 ± 4.16	0.662

**EOB operation skills (0–100), mean ± SD**	42.68 ± 11.75	43.21 ± 12.08	0.752

**Self-efficacy (10–50), mean ± SD**	25.93 ± 6.42	26.15 ± 6.78	0.819


Note: SD = standard deviation; OR = operating room; EOB = electric operating bed.

### Primary Outcome: EOB Operation Skills

The mixed-design ANOVA revealed a significant time × group interaction effect on EOB operation skills (F[2, 796] = 124.57, p < 0.001, partial η² = 0.39), indicating that the pattern of change in EOB operation skills over time differed between the two groups. There were also significant main effects of time (F[2, 796] = 562.83, p < 0.001, partial η² = 0.74) and group (F[1, 398] = 87.42, p < 0.001, partial η² = 0.31).

Post-hoc analyses indicated that there was no significant difference in EOB operation skills between the two groups at baseline (mean difference = 0.53, 95% CI: –2.76 to 3.82, p = 0.752). However, the experimental group demonstrated significantly higher EOB operation skills than the control group at both post-intervention (mean difference = 18.74, 95% CI: 15.92 to 21.56, p < 0.001, Cohen’s d = 1.65) and follow-up (mean difference = 17.25, 95% CI: 14.37 to 20.13, p < 0.001, Cohen’s d = 1.52).

**Figure 1:**
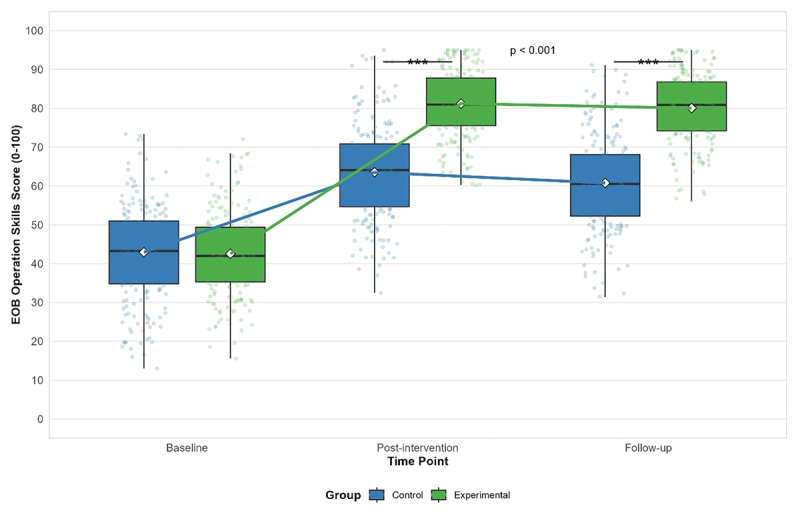
Changes in EOB operation skills scores over time for the experimental and control groups. Box plots show median (horizontal line), mean (diamond), interquartile range (box), and range (whiskers) with individual data points. ***p < 0.001 for between-group comparisons at post-intervention and follow-up.

Within-group comparisons showed that both groups experienced significant improvements in EOB operation skills from baseline to post-intervention (experimental group: mean difference = 38.63, 95% CI: 36.24 to 41.02, p < 0.001; control group: mean difference = 19.89, 95% CI: 17.50 to 22.28, p < 0.001). However, while the experimental group maintained their skills at follow-up (mean difference between post-intervention and follow-up = 1.37, 95% CI: –0.95 to 3.69, p = 0.378), the control group showed a significant decline (mean difference between post-intervention and follow-up = 2.86, 95% CI: 0.54 to 5.18, p = 0.011). Figure 1 illustrates the changes in EOB operation skills over time for both groups.

### Secondary Outcomes

#### Self-efficacy

The analysis of self-efficacy revealed patterns similar to those observed for EOB operation skills. The mixed-design ANOVA showed a significant time × group interaction effect (F[2, 796] = 97.25, p < 0.001, partial η² = 0.33), as well as significant main effects of time (F[2, 796] = 421.68, p < 0.001, partial η² = 0.68) and group (F[1, 398] = 72.16, p < 0.001, partial η² = 0.27).

Post-hoc analyses indicated no significant difference in self-efficacy between the two groups at baseline (mean difference = 0.22, 95% CI: –1.65 to 2.09, p = 0.819). However, the experimental group reported significantly higher self-efficacy than the control group at both post-intervention (mean difference = 9.63, 95% CI: 8.21 to 11.05, p < 0.001, Cohen’s d = 1.79) and follow-up (mean difference = 8.95, 95% CI: 7.48 to 10.42, p < 0.001, Cohen’s d = 1.66).

**Figure 2:**
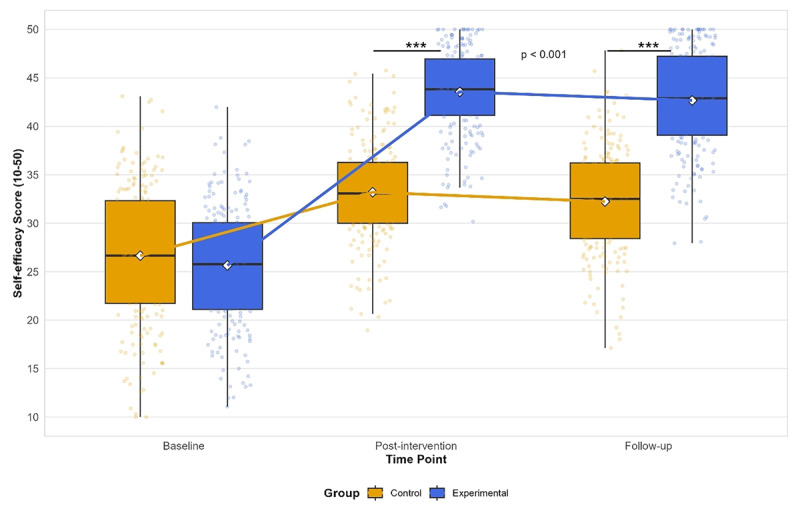
Changes in self-efficacy scores over time for the experimental and control groups. ***p < 0.001 for between-group comparisons at post-intervention and follow-up.

Both groups showed significant improvements in self-efficacy from baseline to post-intervention (experimental group: mean difference = 17.52, 95% CI: 16.25 to 18.79, p < 0.001; control group: mean difference = 7.89, 95% CI: 6.62 to 9.16, p < 0.001). Similar to the pattern observed for EOB operation skills, the experimental group maintained their self-efficacy at follow-up (mean difference between post-intervention and follow-up = 0.73, 95% CI: –0.31 to 1.77, p = 0.241), while the control group showed a slight but significant decline (mean difference between post-intervention and follow-up = 1.41, 95% CI: 0.37 to 2.45, p = 0.005). Figure 2 shows the changes in self-efficacy over time for both groups.

#### Satisfaction

**Figure 3:**
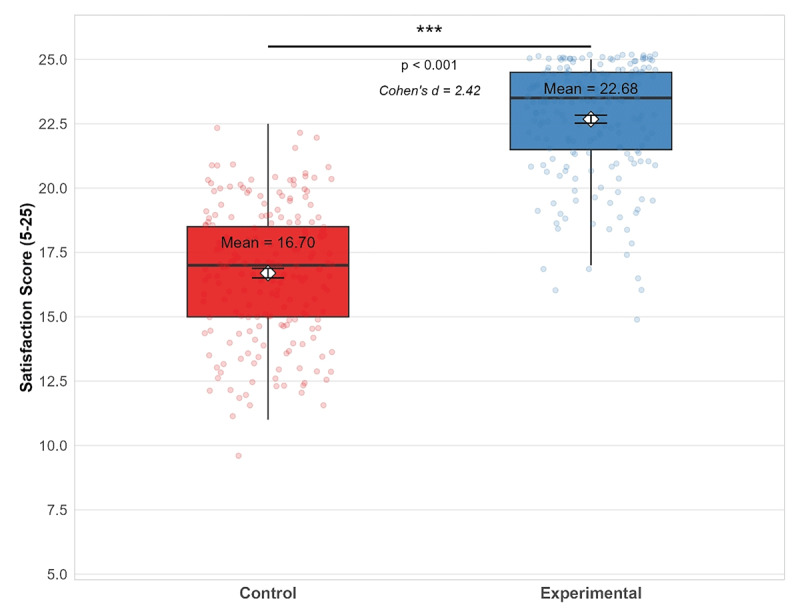
Comparison of satisfaction scores between the experimental and control groups at post-intervention. ***p < 0.001. Note: Higher scores indicate greater satisfaction. Diamond shapes indicate mean values.

The independent t-test revealed that the experimental group reported significantly higher satisfaction with the training than the control group (experimental group: 22.76 ± 2.18; control group: 16.89 ± 2.67; mean difference = 5.87, 95% CI: 5.12 to 6.62, p < 0.001, Cohen’s d = 2.42). Figure 3 presents the comparison of satisfaction scores between the two groups.

### Predictors of EOB Operation Skills Improvement

Multiple linear regression analysis was conducted to identify factors that predicted EOB operation skills improvement. The overall model was significant (F[7, 392] = 37.25, p < 0.001, R² = 0.576), explaining 57.6% of the variance in EOB operation skills improvement.

As shown in Table 2, participation in the experimental group was the strongest predictor of EOB operation skills improvement (β = 0.608, p < 0.001), followed by years of working experience in the operating room (β = 0.192, p < 0.001) and baseline self-efficacy (β = 0.145, p = 0.006). Baseline EOB operation skills were negatively associated with improvement (β = –0.179, p = 0.001), indicating that participants with lower baseline skills showed greater improvement. Age, gender, and education level were not significant predictors.

**Table 2:** Multiple Linear Regression Analysis for Predictors of EOB Operation Skills Improvement.


PREDICTOR	B	95% CI	β	P-VALUE

Group (experimental vs. control)	18.37	15.63, 21.11	0.608	<0.001

Age (years)	0.06	–0.16, 0.28	0.026	0.574

Gender (female vs. male)	–0.92	–4.46, 2.62	–0.022	0.610

Education level (higher vs. lower)	0.81	–1.67, 3.29	0.029	0.520

Years of working experience in OR	0.73	0.39, 1.07	0.192	<0.001

Baseline self-efficacy	0.34	0.10, 0.58	0.145	0.006

Baseline EOB operation skills	–0.23	–0.37, –0.09	–0.179	0.001


Note: CI = confidence interval; OR = operating room; EOB = electric operating bed.

### Relationship Between Self-efficacy and EOB Operation Skills

**Figure 4:**
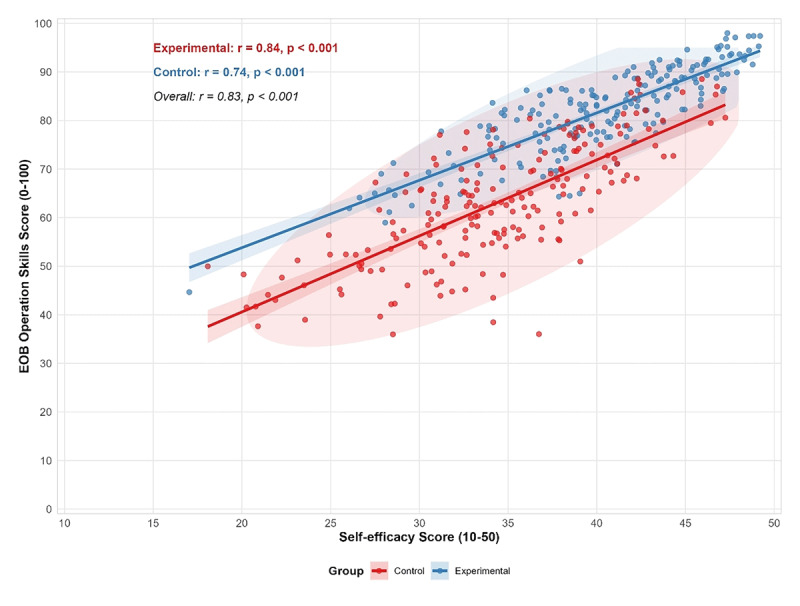
Correlation between self-efficacy and EOB operation skills at post-intervention for the experimental (blue) and control (red) groups, with regression lines and 95% confidence intervals.

Pearson’s correlation analysis revealed a strong positive correlation between self-efficacy and EOB operation skills at post-intervention (r = 0.83, p < 0.001) and follow-up (r = 0.80, p < 0.001). This correlation was stronger in the experimental group (post-intervention: r = 0.84, p < 0.001; follow-up: r = 0.82, p < 0.001) than in the control group (post-intervention: r = 0.74, p < 0.001; follow-up: r = 0.73, p < 0.001). Figure 4 illustrates the correlation between self-efficacy and EOB operation skills at post-intervention for both groups.

### ROC Curve Analysis

ROC curve analysis was performed to determine the optimal cut-off value of self-efficacy for predicting successful acquisition of EOB operation skills (defined as achieving at least 80% of the maximum score on the EOB operation skill test at post-intervention). The area under the curve (AUC) was 0.9000 (95% CI: 0.87 to 0.95, p < 0.001), indicating excellent discriminative ability.

**Figure 5:**
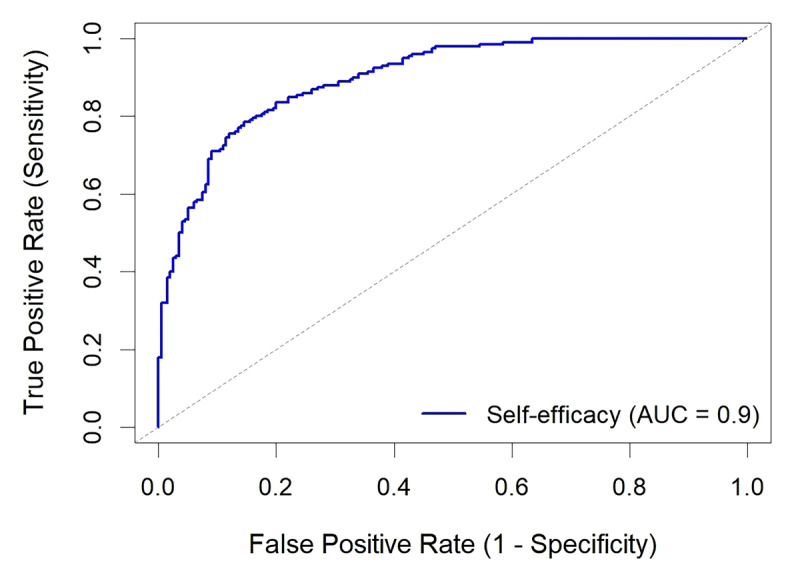
Receiver operating characteristic (ROC) curve for self-efficacy in predicting successful acquisition of EOB operation skills (AUC = 0.9).

The optimal cut-off value was identified as 39 points on the self-efficacy scale, which provided a sensitivity of 86.5% and a specificity of 82.3%. This suggests that participants with a self-efficacy score of 39 or higher had a high probability of successfully acquiring EOB operation skills. Figure 5 presents the ROC curve for self-efficacy in predicting successful acquisition of EOB operation skills.

### Sensitivity Analysis

The sensitivity analysis comparing the results of the complete-case analysis with those of the multiple imputation analysis showed consistent findings, indicating that the missing data did not substantially influence the results. The effect sizes and significance levels were similar in both analyses, supporting the robustness of the findings.

## Discussion

This randomized controlled trial investigated the effectiveness of the BL-BOPPPS model in EOB training for operating room nurses compared to traditional training methods. The findings demonstrate that the BL-BOPPPS approach is associated with significantly greater improvements in EOB operation skills, self-efficacy, and satisfaction than traditional training, with these benefits maintained at three-month follow-up. These results provide evidence supporting the value of the BL-BOPPPS model in technical skills training for specialized nursing roles, offering important implications for nursing education and practice.

The substantial improvement in EOB operation skills observed in the experimental group is consistent with the theoretical framework outlined in the introduction. As described, the BL-BOPPPS model was designed on the basis of constructivist learning theory, which emphasizes the active construction of knowledge through meaningful experiences and reflection [9,10]. The structured learning process—including explicit connections to prior knowledge (Bridge-in), clear learning objectives (Learning Outcomes), baseline assessment (Pre-assessment), active engagement (Participatory learning), outcome evaluation (Post-assessment), and synthesis (Summary)—operationalized these constructivist principles into a comprehensive instructional framework. The observed improvements suggest that this theory-guided design was effective in enhancing skill acquisition in the EOB training context.

The participatory component of the BL-BOPPPS model also aligns with critical reflection principles, as described by Thompson and Thompson [24], who emphasize that reflective practice enhances professional development by encouraging learners to analyze experiences and integrate new knowledge with existing understanding. This process of critical reflection was facilitated through the pre-assessment and post-assessment phases of the BOPPPS model, allowing participants to identify gaps in their knowledge and skills and to develop strategies for improvement.

As articulated in the introduction, the participatory component of the BL-BOPPPS model was explicitly informed by Vygotsky’s sociocultural theory and the concept of the zone of proximal development [11]. The structured instructor demonstrations, peer interactions, and graduated feedback during the participatory learning phase were designed to scaffold learning within participants’ ZPD. The observed effectiveness of this approach—particularly the large effect sizes for skills improvement—is consistent with BL-BOPPPS mode theoretical perspective and supports the value of socially mediated learning in clinical skills training.

The findings of this study have broader implications for nursing practice environments beyond the operating room. White et al. [25] found that work environment significantly impacts care quality and nurse outcomes, including burnout and job satisfaction. By enhancing technical competence and self-efficacy through effective educational approaches like the BL-BOPPPS model, healthcare organizations may potentially improve nurse satisfaction and reduce burnout, particularly in high-stress environments like operating rooms. Future research should examine whether improvements in training methodology translate to enhanced workplace satisfaction and retention among nursing staff.

The blended learning aspect of the intervention also contributed to its effectiveness by providing flexible access to learning resources and opportunities for self-paced review. This component addresses the needs of adult learners for autonomy and self-direction, as highlighted by adult learning theory [12,13]. The combination of online resources and face-to-face instruction created multiple pathways for knowledge acquisition and reinforcement, potentially enhancing the depth and retention of learning. This finding is consistent with previous research by Yin and Yuan [26], who found that blended learning approaches could effectively detect and address learning gaps through topic modelling.

The significant improvement in self-efficacy observed in the experimental group represents another important outcome of the BL-BOPPPS intervention. Self-efficacy, defined as one’s belief in one’s ability to succeed in specific situations, has been identified as a crucial factor in performance across various domains [22]. The strong correlation between self-efficacy and EOB operation skills found in this study suggests that the BL-BOPPPS model not only enhanced technical skills but also strengthened participants’ confidence in their abilities. This dual impact on skills and self-efficacy is particularly valuable in clinical nursing education, where both technical competence and professional confidence are essential for effective practice.

The ROC curve analysis, which identified a self-efficacy cut-off score that predicted successful acquisition of EOB operation skills with high sensitivity and specificity, provides a practical tool for educators. This threshold could be used to identify learners who may require additional support or intervention during training. By monitoring self-efficacy throughout the training process, educators could potentially identify at-risk learners early and implement targeted interventions to enhance both their confidence and skills.

The regression analysis identified several significant predictors of EOB operation skills improvement, with participation in the BL-BOPPPS training emerging as the strongest predictor. This finding reinforces the value of this pedagogical approach for technical skills training in nursing education. The positive association between years of working experience and skills improvement suggests that the BL-BOPPPS model effectively leverages existing clinical knowledge, allowing more experienced nurses to build on their foundation. This aligns with the constructivist perspectives described in the introduction, which emphasize the importance of connecting new information to existing knowledge structures [9,10].

The negative association between baseline skills and improvement indicates that the BL-BOPPPS model was particularly beneficial for participants with lower initial skill levels. This finding has important implications for educational practice, suggesting that this approach may be especially valuable for addressing skill deficits and reducing performance gaps among learners. The structured nature of the BOPPPS model, with its emphasis on baseline assessment and targeted instruction, may be particularly effective for identifying and addressing individual learning needs.

The high satisfaction levels reported by participants in the experimental group suggest that the BL-BOPPPS model was well-received by learners. This positive reception is crucial for the practical implementation of educational innovations, as learner engagement and acceptance significantly influence the success of training programs. The comprehensive structure of the BOPPPS model, which addresses various aspects of the learning process, may have contributed to this positive reception by providing a clear framework for instruction and assessment.

The maintenance of skills and self-efficacy at three-month follow-up in the experimental group, contrasted with the decline observed in the control group, suggests that the BL-BOPPPS model promotes better retention of learning. This finding has important implications for clinical practice, where the sustainability of training effects is crucial for long-term performance improvement. The active engagement, repeated practice, and explicit connections to clinical applications emphasized in the BL-BOPPPS approach may have facilitated deeper processing and stronger memory consolidation, contributing to better retention.

These findings align with previous research on the BL-BOPPPS model in other educational contexts. Ma et al. [18] found that implementing the BL-BOPPPS model in health services management education resulted in improved student outcomes and greater learner satisfaction compared to conventional teaching methods. Similarly, Dai et al. [19] reported enhanced knowledge acquisition and critical thinking skills among veterinary medicine students taught using the BL-BOPPPS approach. The present study extends these findings to nursing education, specifically to technical skills training for operating room nurses, demonstrating the broader applicability of this pedagogical model across healthcare education domains.

The implications of this study for nursing education and practice are substantial. First, the findings suggest that the BL-BOPPPS model represents a valuable approach for technical skills training in nursing education, offering a structured yet flexible framework that can be adapted to various learning contexts. The clear delineation of instructional components within the BOPPPS model provides a practical guide for educators seeking to enhance the effectiveness of their teaching.

Second, the study highlights the importance of active, participatory learning experiences in nursing education. The significant performance differences between the experimental and control groups underscore the limitations of traditional, lecture-based approaches for skills development and the value of interactive, experiential learning strategies. This finding challenges traditional educational practices in nursing and supports the shift toward more learner-centered, active approaches.

Third, the strong relationship between self-efficacy and performance identified in this study emphasizes the importance of addressing both cognitive and affective dimensions of learning in nursing education. Training programs should aim not only to develop technical skills but also to foster learners’ confidence in their abilities, recognizing the reciprocal relationship between competence and confidence.

Finally, the sustained benefits observed at follow-up in the experimental group suggest that the BL-BOPPPS approach may contribute to more durable learning outcomes, potentially reducing the need for frequent refresher training and enhancing the efficiency of educational programs. This has implications for resource allocation and program planning in nursing education and staff development.

Despite its strengths, this study has several limitations that should be considered. First, the intervention was implemented in a single institution with a relatively homogeneous sample of ORNs in one city in China, potentially limiting the generalizability of the findings to other settings, populations, or healthcare systems. Future research should include multiple sites and diverse participant groups across different regions and countries to enhance external validity.

Second, while the study compared the BL-BOPPPS model to traditional didactic training, it did not compare the BL-BOPPPS approach to other participatory, constructivist instructional models, such as the 4C/ID model [5], problem-based learning, or team-based learning. Therefore, the observed improvements cannot be attributed specifically to the unique features of the BOPPPS framework as opposed to the general benefits of active, participatory learning. Future research should include comparisons with other evidence-based instructional designs to isolate the specific contributions of the BOPPPS structure.

Third, the experimental and control conditions differed in several ways beyond the pedagogical model, including access to asynchronous online materials (additional time on task), structured opportunities for hands-on practice, and systematic feedback from instructors and peers. Each of these elements—learning from feedback, test-enhanced learning, and increased time on task—has been shown independently to enhance learning outcomes [27,28]. The present design does not allow us to disentangle the relative contributions of these individual components from the overall effect of the BL-BOPPPS model. Future studies employing factorial or dismantling designs would be valuable for identifying which specific elements drive the observed improvements.

Fourth, the follow-up period was relatively short (three months), limiting our understanding of the long-term retention of skills and self-efficacy. Longer follow-up periods would provide more robust evidence regarding the durability of the training effects and potentially reveal patterns of skill decay over time.

Fifth, while the study measured EOB operation skills, self-efficacy, and satisfaction, it did not assess the transfer of these skills to clinical practice or their impact on patient outcomes. Future research should examine the relationship between training outcomes and clinical performance, including patient safety indicators and workflow efficiency.

Sixth, the study did not include a qualitative component to explore participants’ experiences with the BL-BOPPPS model. Mixed-methods research combining quantitative measures with qualitative insights would provide a more comprehensive understanding of the educational processes involved. It should also be noted that, while we describe the associations between the BL-BOPPPS model and higher satisfaction and self-efficacy, these outcomes are better understood as correlates of the intervention rather than mechanisms explaining how the intervention enhanced learning.

Finally, while the study compared the BL-BOPPPS model to traditional training, it did not isolate the effects of the blended learning component from the BOPPPS framework itself. Future research could employ a factorial design to examine the individual and combined effects of these components, providing more nuanced insights into the elements that drive educational effectiveness.

In conclusion, this study provides evidence for the effectiveness of the BL-BOPPPS model in EOB training for operating room nurses. The significant improvements in EOB operation skills, self-efficacy, and satisfaction, along with the maintenance of these benefits at follow-up, suggest that this pedagogical approach represents a valuable innovation for nursing education. By integrating structured instructional design with blended learning strategies, the BL-BOPPPS model offers a comprehensive framework for enhancing technical skills training in specialized nursing roles. While these findings are encouraging, future research comparing the BL-BOPPPS model with other participatory instructional approaches and conducted across multiple sites is needed to further establish the generalizability and specificity of its effectiveness. The findings of this study support the broader implementation of this approach in nursing education and highlight directions for future research to further refine and extend its application.

## Data Availability

All data generated or analyzed during this study are included in this published article.
